# Challenging 10 misconceptions in conservation physiology

**DOI:** 10.1093/conphys/coag030

**Published:** 2026-05-08

**Authors:** Christine L Madliger, Nann A Fangue, Kathleen Hunt, Edward Narayan, Cosima S Porteus, Jennifer F Provencher, Jodie L Rummer, Frank Seebacher, Anne E Todgham, Sean Tomlinson, Essie Rodgers, Craig E Franklin, Steven J Cooke

**Affiliations:** Department of Biology, Algoma University, 1520 Queen St. E., Sault Ste. Marie, Ontario P6A 2G4, Canada; Department of Wildlife, Fish, and Conservation Biology, University of California Davis, 1 Shields Ave., Davis, CA 95616, USA; Smithsonian-Mason School of Conservation and George Mason University, 1500 Remount Rd., Front Royal, VA 22630, USA; School of Agriculture and Food Sustainability, Faculty of Science, The University of Queensland, 5391 Warrego Highway, Gatton, QLD 4072, Australia; Faculty of Science and Engineering, Southern Cross University, Military Rd., East Lismore, New South Wales 2480, Australia; Department of Biological Sciences, University of Toronto Scarborough, 1265 Military Trail, Toronto, Ontario M1C 1A4, Canada; National Wildlife Research Centre, Environment and Climate Change Canada, 1125 Colonel By Drive, Raven Road, Ottawa, Ontario K1S 5B6, Canada; Marine Biology, College of Science and Engineering, James Cook University, 1 James Cook Dr., Townsville, QLD 4811, Australia; School of Life and Environmental Sciences A08, University of Sydney, New South Wales 2006, Australia; Department of Animal Science, University of California Davis, 1 Shields Ave., Davis, CA 95616, USA; School of Molecular and Life Sciences, Curtin University, Kent Street, Bentley, Perth, Western Australia 6102, Australia; School of Biological Sciences, The University of Adelaide, North Terrace, Adelaide, South Australia 5000, Australia; School of Environmental and Conservation Sciences, Murdoch University, 90 South Street, Murdoch, Western Australia 6150, Australia; School of the Environment, The University of Queensland, Brisbane QLD 4072, Australia; Department of Biology and Institute of Environmental and Interdisciplinary Science, Carleton University, 1125 Colonel By Dr Ottawa, Ottawa, Ontario K1S 5B6, Canada

**Keywords:** Baseline data, criticism, ecophysiology, non-invasive, opportunities, solutions, surrogate species, toolbox

## Abstract

Relative to other subdisciplines of conservation science, conservation physiology remains somewhat nascent. Although there is a growing number of successes where physiological concepts, knowledge and tools have informed conservation decisions and management actions, there also remain a number of challenges. We argue that there is a set of misconceptions that serve as *de facto* barriers to realizing the full potential of conservation physiology as a mission-oriented area of scholarly inquiry and practice. However, we also suggest that those misconceptions can be easily dispelled. In this paper, we identify and dispel 10 ‘myths’ in conservation physiology: (i) conservation physiology subsumes other disciplines and has no unique goals; (ii) the toolbox is too invasive; (iii) tools are too specialized; (iv) the field relies too heavily on surrogates and captive studies; (v) physiological information cannot be scaled from the individual level to the population level; (vi) baseline (i.e. control) data are too sparse; (vii) conservation physiology is too speculative; (viii) managers do not care about physiology; (ix) conservation physiology cannot engage fundamental scientists; and (x) conservation physiology cannot solve conservation problems. Given that some of these misconceptions represent ongoing barriers, we also identify opportunities for further overcoming them. In particular, embracing co-production and engaging in effective knowledge exchange are fundamental to generating relevant and actionable knowledge. Similarly, we submit that the tension between fundamental and applied science is not inherently disruptive and ensures that there is a strong foundation for evidence-based decisions. This paper is intended to open the eyes and minds of ecologists, physiologists and end-users about what conservation physiology has to offer and how to realize those opportunities. Working collectively and collaboratively to achieve the common mission of conservation physiology will not only benefit conservation science but will also benefit biodiversity and society.

## Introduction

*Conservation Physiology* has been in existence for over a dozen years, publishing its inaugural issue in 2013. Since its launch, the journal has become a venue for progressing the field in tangible ways; it has promoted the validation and refinement of physiological tools (Madliger *et al.*, 2018), synthesized key themes surrounding the application of physiological information to conservation science (e.g. translocations: Tarszisz *et al.*, 2014; invasive species: Shaik *et al.*, 2016; environmental protection programmes: Glover, 2018; captive breeding: Silla *et al.*, 2021; climate warming: Seebacher *et al.*, 2023) and provided evidence that a diversity of techniques, taxa and conservation challenges fall under the umbrella of conservation physiology (Cooke *et al.*, 2013; Coristine *et al.*, 2014). Despite the growth of the conservation physiology community and the associated success stories (Madliger *et al.*, 2016; Madliger *et al.*, 2020; Cooke *et al.*, 2025), the discipline still faces criticism related to its value within the larger conservation science toolbox. For example, during the collection of data using an online survey of scientists who have published work combining conservation science and physiology, Madliger *et al.* (2021a) received open-ended comments questioning the value of the field in relation to the invasiveness of its tools, uniqueness of its goals and its accessibility to managers and decision-makers (Box 1). As a diverse set of conservation physiologists from around the globe who use different approaches/tools and focus on various taxa and questions, we have been privy to similar criticisms raised at conferences and meetings, through reviewer comments and during other interactions with colleagues and partners.

*Conservation Physiology*’s inaugural paper (Cooke *et al.*, 2013) summarized the goals and scope of the field at that time, and many contributions that have followed share a strong overtone of optimism for opportunities and successes in the future. There have since been a variety of reviews, perspectives and empirical studies that have highlighted the benefits of physiological approaches for conservation science, including those focused on horizon scans (Cooke *et al.*, 2020), summaries of success stories (Madliger *et al.*, 2016), opportunities to promote evidence-based decision-making (Cooke *et al.*, 2017), improving translocation strategies (Tarszisz *et al.*, 2014), determining climate change vulnerability (Khaliq *et al.*, 2014; Evans *et al.*, 2015; Bennett *et al.*, 2019), ecological risk assessments (Glover, 2018), endangered species recovery (Birnie-Gauvin *et al.*, 2017; Mahoney *et al.*, 2018; Madliger *et al.*, 2022), predicting disease dynamics (Rohr *et al.*, 2013; Hing *et al.*, 2016; Ohmer *et al.*, 2021) and managing invasive species (Funk, 2013; Lennox *et al.*, 2015), among many others. Here, we aim to continue this momentum by dispelling 10 common ‘myths’ related to conservation physiology. These 10 misconceptions were formalized through input from our authorship team and informed by the criticisms highlighted in Box 1. We provide an overview of each criticism, outline evidence to the contrary and, where relevant, present ways to further work towards addressing the challenge. These themes collectively reflect the complex landscape surrounding the integration of physiology and conservation science, highlighting both challenges and opportunities for the field. We end with a discussion of the importance of co-production for linking physiological information with conservation action and provide a list of resources that can act as a starting point for those considering the use of physiology in their systems for the first time.

We acknowledge that many of the misconceptions we cover are not unique to the discipline of conservation physiology and that other fields have faced them during their development and evolution. However, we argue that this commonality is not a valid reason to dismiss them. Reflecting on available evidence and collating counter-arguments can improve our understanding of the state of these challenges within the context of conservation physiology, allowing individuals working in the field to more cogently discuss them with colleagues or potential partners/collaborators. We see any opportunity to progress past some of these ‘growing pains’ more quickly as wholly positive and as a way to increase confidence in the field, particularly in those who have not engaged with its tools or applications. Given that no discipline can independently solve the vast array of conservation challenges being faced across the globe (Game *et al.*, 2014), finding ways to enhance the application of underutilized disciplines like conservation physiology can leave us better equipped to implement gains for conservation.

## Ten misconceptions addressed

### Conservation physiology subsumes other disciplines and has no unique goals of its own

Although conservation physiology has grown to yield symposia, a journal, textbook volumes (Cooke *et al.*, 2022; Fangue *et al.*, 2022), a full textbook (Madliger *et al.*, 2020) and university-level courses on the topic, there remain questions about the uniqueness of the discipline and whether it warrants being categorized as independent from other conservation-focused fields. While difficult to pinpoint, there seem to be two parallel areas of development behind conservation physiology (Madliger *et al.*, 2021b). The first is ecotoxicology, with some of the work done in the middle of the 20th century that identified mechanistic pathways of effect between contaminants and fitness leading to regulatory changes (Zhou *et al.*, 2018). The second is the efforts related to applied endocrinology (sometimes termed ‘conservation endocrinology’; Schoech and Lipar, 1998; McCormick and Romero, 2017), which initially grew out of reproductive physiology focused on captive breeding (Holt *et al.*, 2003) and has since expanded to monitoring animal condition (e.g. stress and reproductive state) in the wild (Walker *et al.*, 2005). The last decade has seen the emergence of a third area of interest incorporating ‘functional traits’ (principally energetics and thermal biology) with spatiotemporal modelling (Kearney *et al.*, 2010) as a result of growing computational capacity to model plausible future conservation scenarios. Cooke *et al.* (2013) classifies these domains under the term ‘conservation physiology’ but in doing so does not erase the important work done previously or since. Conservation physiology today is more holistic than both of those original domains, and it brings together physiologists that work on different systems (including non-animal systems) and levels of biological organization (Bergman *et al.*, 2019). Currently, conservation physiology makes both fundamental contributions to our understanding of biological mechanisms and generates knowledge directly and immediately relevant to conservation (as per its definition; Cooke *et al.*, 2013). However, as a discipline that is maturing, it extends further in terms of developing a community of diverse professionals committed to training the next generation, creating inclusive spaces (Cooke *et al.*, 2020) and ensuring conservation physiology contributes to meaningful conservation outcomes (Cooke and O'Connor, 2010; Madliger *et al.*, 2016, 2017).

Disciplines are not static; there is a constant pattern of convergence and divergence that reflects disciplinary evolution. Even longstanding disciplines may change over time or disappear as they are replaced with others. When there are complex problems that cannot be solved by a single discipline, it is not uncommon for new interdisciplinary domains of scholarly activity to be defined; this process well-captures what we have seen in conservation science over the last ~40 years (Soulé, 1985; Caughley, 1994). As conservation science (initially termed conservation biology but since broadened to conservation science to reflect breadth) evolved, a variety of subdisciplines proliferated (e.g. conservation behaviour, conservation genetics, conservation social science and conservation biogeography), each with its own subculture of conferences, journals and courses, and each with its own degree of recognition as an ‘independent’ discipline. Each of these disciplines shares the same ultimate goal: to solve conservation challenges. Their divergence lies in the tools and approaches that they use to do so and the circumstances in which they can best be applied. Therefore, at minimum, each has its own evolving goals to improve the associated tools and bolster the underlying theories associated with their use. Conservation physiology has its own goals in that it is focused on understanding and solving conservation problems specifically through a consideration of mechanisms (Wikelski and Cooke, 2006; Cooke *et al.*, 2013). Although there are some shared overarching objectives and approaches, many of the goals of conservation physiology as defined by Cooke *et al.* (2013) (e.g. understanding the relevance of ecology and evolution of physiological diversity to conservation, exploiting knowledge of organismal physiology to control invasive species and restore threatened habitats and populations, applying physiological biomarkers as part of long-term environmental monitoring programmes, developing predictive models in conservation practices that include physiological parameters, integrating physiological knowledge into ecosystem management and development of tools to solve complex conservation problems, etc.) are not covered by any other subdiscipline in conservation science. To that end, we submit that conservation physiology is itself a valid and important discipline that comprises a unique subdiscipline within conservation science. It is mission oriented and is delivering on its promise in ways that could not be achieved by thinking about biological systems in less holistic ways. Like all disciplines, it will grow most effectively when researchers and practitioners reflect on its overall progress, breadth, successes and failures to allow for goal refinement and expansion.

### The conservation physiology toolbox is too invasive

Wildlife physiological studies initially acquired a reputation of being ‘invasive’ due to the fact that they drew on the tools of classical laboratory physiology, which often require restraint of the animal for tissue sampling or other procedures. Thus, an early focus of conservation physiology (prior to it being defined in 2006; Wikelski and Cooke, 2006) was to reduce the invasiveness of the classic physiology toolkit. One of the first widespread efforts at developing non-invasive tools involved faecal hormone assays, with early validation studies in the 1980s focusing on progesterone (for monitoring mammalian pregnancy) and cortisol (for monitoring ‘stress’). Some 20 years were then spent in validating these assays and grappling with the numerous issues that faeces pose as a sample type (Touma and Palme, 2005), but for the most part, these issues have been resolved satisfactorily. In the last two decades this toolkit has been progressively broadened to include numerous other analytes including aldosterone, thyroid hormones, immunoglobulins, dietary markers and toxins, with new biomarkers constantly under investigation (Madliger *et al.*, 2018). More recent expansion of this work has also led to the proliferation of the use of several fields of -omics studies (metabolomics, proteomics, etc.), which are the wide-scale studies of groups of physiological molecules and how they interact. Moreover, other biological matrices including keratin tissues (feather, fur, claw, etc.; Whitehead and Dunphy, 2022; Carbajal *et al.*, 2024; Samsonova *et al.*, 2025) and respiratory vapour (including collection by drones; O'Mahony *et al.*, 2024) have been validated, creating more opportunities for non-invasive monitoring (see Pirotta *et al.*, 2023). Other innovations with technology such as electronic tags (biologgers and biotelemetry; Wilson *et al.*, 2015; Barham *et al.*, 2025), respiration and heart rate measured via infrared thermal imagery (Rzucidlo *et al.*, 2023), drone-based metrics of body condition (Bierlich *et al.*, 2025) and field respirometers (Mochnacz *et al.*, 2017) have expanded the toolbox even more.

Innovative methodologies and commitment to refinement have, in some cases, allowed for physiological studies despite the imperilled status of study species. For example, clonal propagation of the critically endangered Wollemi pine (*Wollemia nobilis*) has allowed for investigations into growth and stem development, metabolism, pathogens and wound healing and photosynthetic rates without putting pressure on the remnant population (Trueman *et al.*, 2007; Heady and Burrows, 2008; Offord *et al.*, 2014; Lewis *et al.*, 2015; Giebelhaus *et al.*, 2023). Overall, non-invasive physiological monitoring has been employed in imperilled species across taxa (e.g. western quoll [*Dasyurus geoffroi*; Jensen *et al.*, 2019]; Yangtze finless porpoise [*Neophocaena asiaeorientalis asiaeorientalis*; Hao *et al.*, 2019]; whooping cranes [*Grus americana*; Fitzpatrick *et al.*, 2015]; loggerhead turtles [*Caretta caretta*; Sakamoto *et al.*, 2021]; Fijian ground frog [*Platymantis vitiana*; Narayan *et al.*, 2010]). These examples are not comprehensive but serve to illustrate that there is both opportunity and ongoing efforts for refining the conservation physiology toolbox to employ non-invasive and minimally invasive tools. This is, of course, useful from an animal welfare perspective (Zemanova, 2020; Lane and McDonald, 2024) but, in the context of conservation, is particularly salient due to concerns with using invasive methods on rare and imperilled species (Paquet and Darimont, 2010; McMahon *et al.*, 2012). We encourage the dissemination of ‘toolbox’ or methodological papers that highlight the changing technological landscape of physiological monitoring, such as miniaturization, validation and measurement accuracy (Lennox *et al.*, 2025), thereby calling to attention the increasing accessibility of low- and non-invasive tools.

### Without specialized expertise, conservation physiology tools are out of reach

Advances in technology are often cited for their power in allowing more detailed measurements of physiological responses and spatiotemporal interactions between the organism and the environment (Cooke *et al.*, 2004; Tomlinson *et al.*, 2014). It is undeniably true that some of the advanced technology available, such as metabolomics, biologgers or stable isotope analyses, is costly and requires unique skills or experience to be used to best effect, and the data that result can seem obscure. Furthermore, the statistical analyses of these data are increasingly complex and often require substantial computing power that may be well beyond the capacity of non-government organizations, especially in the developing world or other under-sourced regions. Even with physiological metrics that have been measured for decades in conservation contexts (e.g. glucocorticoids), proper interpretation requires an understanding of context dependency, pleiotropy and the larger functioning of the endocrine stress axis (MacDougall-Shackleton *et al.*, 2019; Vitousek *et al.*, 2019). These characteristics have led to criticisms of conservation physiology as being too complex, too expensive and too impenetrable to provide practical guidance to grassroots conservation. At its heart, however, ecological physiology is intrinsically focused on cause and effect: the cause being changes in the environment and the effect being the performance of the organism (Bradshaw, 2003). Viewed in this framework, some data are generally quite intuitive, even without specialized training in the techniques or technologies being used. It should also be borne in mind that physiological traits were some of the first to be measured by science; for example, Lavoisier and LaPlace were measuring metabolic rate by calorimetry in 1783 (Morris, 1972), and a simple respirometer proposed over 50 years ago (Gilson, 1963) is still in use today.

While a lot of physiological research is undertaken by direct measurement of physiological processes such as endocrine changes or energetic fluxes, a great deal of insight can be gained by measuring performance analogues. For example, many studies of thermal tolerance have been structured on the basis of the performance of the whole organism [e.g. locomotor performance (Cabezas-Cartes *et al.*, 2019), germination success (Rajapakshe *et al.*, 2022); fecundity (Becheler *et al.*, 2022)]. Though it is true that many physiological studies apply highly technical tools to measure subtle and detailed physiological responses to environmental changes, this is not essential to gain substantial conservation insights that might result from physiological research. Indeed, a strong argument has been made that simple research questions, integrated with an applied focus, can make very powerful contributions to conservation programmes (Young, 2000). This concept is no less true for conservation physiology than for any other discipline. In circumstances where specialized input is necessary, it should be noted that many successful conservation physiology projects involve teams of researchers and practitioners with expertise that collectively allows for the tackling of complex conservation problems (see examples in Madliger *et al.*, 2020 and Cooke *et al.*, 2025). In this way, there are opportunities to work collaboratively to collect, analyse and interpret physiological data. We urge participants to engage in these partnerships early in the planning stages, as many of the complexities of interpreting physiological data can be addressed with sound experimental design and attention to methodological details. We discuss co-production in more detail and describe it as an essential approach for realizing the full potential of conservation physiology in the final section.

### Conservation physiology relies too heavily on surrogates and captive studies

One common criticism of conservation physiology stems from its origins in conservation biology *sensu lato*, which is its apparent preoccupation with endangered species and small populations (Caughley, 1994). By extension, if each species’ physiology is truly unique, then it is not possible to study more common species as surrogates of threatened species to direct their conservation. Both points overlook the heritage that conservation physiology has in comparative physiology. The comparative paradigm is fundamentally interested in the effects of ecology and evolution on otherwise generalizable relationships in physiological traits (Somero, 2011). Such an approach inherently uses surrogates to provide phylogenetically informed insights into the probable physiological capacity and tolerance of threatened species, and it provides a robust approach to do so, being informed by large meta-datasets of empirical trait data that have only recently begun to be emulated elsewhere (e.g. PanTHERIA, Jones *et al.*, 2009; Phylacine, Faurby *et al.*, 2018). Physiological responses are at the interface between the environment and organisms and are therefore at the most relevant level of inquiry into the effects of environmental change. Despite the gap between physiological studies and a realistic ecological context, physiological research can transcend the specific to define general responses. Physiological research typically aims to uncover in-principle functional capacities and constraints (Rajapakshe *et al.*, 2022; Cicchino *et al.*, 2023; Monge *et al.*, 2023) rather than documenting instantaneous conditions. Physiological research therefore provides insights that often apply across environments, rather than to specific situations, and physiological data identify environmental boundary conditions within which individuals, populations or species can operate. For example, critical thermal minima and maxima define the ‘safe’ operating conditions for different species (Gunderson and Stillman, 2015), and the thermal performance breadth indicates the range of thermal conditions within which organisms maintain their fitness (Sinclair *et al.*, 2016). These insights are invaluable in assessing potentially suitable habitats in environments that change as a result of human activities. This approach extends to multi-stressor environments where identification of trade-offs in physiological and behavioural responses to different stressors are essential to assess viability of organisms under different regimes of disturbance (Dominoni *et al.*, 2020; Seebacher, 2022; Lopez *et al.*, 2023).

Physiological responses are often conserved phylogenetically, which confers generality to insights gained from model organisms. For example, maintaining a favourable energy budget is closely linked to fitness and any environmental disruption of energy metabolism is likely to be detrimental (Shultz *et al.*, 2021). The infrastructure underpinning energy metabolism is highly conserved among metazoans so that the constraints that operate on a particular model organism are likely to also operate across a broader range of species. The phylogenetic context across which insights from model organisms can be extrapolated can be further refined by knowledge of the evolution of particular physiological systems. For example, signalling systems can be more derived phylogenetically and a regulatory system such as thyroid hormones is most complex in vertebrates among which it is highly conserved (Takagi *et al.*, 2022; Morthorst *et al.*, 2023). Hence, knowledge of the potential conservation implications of environmental thyroid disruption gained from model systems (Lee *et al.*, 2019) is also likely to apply in principle to other vertebrates. Hence, physiology is an important component of conservation by its ability to identify environmental boundaries and constraints, which can be interpreted together with knowledge of the phylogenetic conservatism of physiological systems.

It is noteworthy, however, that the use of surrogate species may be effective at the individual level but not at the population level. For example, the common rainbow (*Oncorhynchus mykiss*) and brown trout (*Salmo trutta*) predicted individual responses of the threatened cutthroat trout (*Oncorhynchus clarkii stoias*) well, but failed to do so at the population level (Accolla *et al.*, 2019). The application of surrogate species may also be limited in very small and isolated populations where trait means shifted away from those predicted by surrogate species as a result of genetic drift (Meek *et al.*, 2022). For populations and species not on the brink of extinction, however, genetic and phenotypic variation among individuals can increase persistence in changing environments (Forsman *et al.*, 2012; Schindler *et al.*, 2015). Increased genetic diversity as a result of local adaptation or plasticity resulting from epigenetic processes can therefore increase the resilience of populations and species in variable environments (Meek *et al.*, 2022; Noble *et al.*, 2025). Surrogate species could be used to estimate individual and population variability, and the resulting data can be a first estimate of likely variation in species that are conservation targets and more difficult to study. Indeed, a meta-analysis indicates that genotypic and phenotypic diversity improves population persistence consistently across plant and animal species (Forsman, 2014), which is encouraging for the use of surrogates. However, to the best of our knowledge, there are no formal applications of surrogates to estimate trait variation, but exploring this approach further could make an important contribution to conservation. Overall, integrating approaches across a laboratory-field continuum will permit balance between measurements in real-world environmental scenarios and highly controlled captive experiments (Binning *et al.*, 2025). These integrative endeavours will provide information essential to refining the application of conservation physiology, including deciphering links between individual physiology and population- or ecosystem-level effects, pinpointing when model organisms best represent their imperilled counterparts and determining how multiple environmental factors interact to elicit physiological responses (Binning *et al.*, 2025).

### Physiological information collected from individuals cannot be scaled to the population level

Conservation in a narrow, biological sense may be defined as actions intended to enhance the chances of habitats and species to persist in the wild (Sandbrook, 2015). The general perception is that conservation is an ecological discipline that operates at the species or higher level of organization (Mangel *et al.*, 1996). Conservation biology therefore has a top-down focus, observing patterns at a large, often macro-ecological scale (e.g. declining species, habitat destruction, reduced biodiversity). The success of many conservation measures depends on the local context, the particular species to be protected or the characteristic of the protected area, and the specific conservation action does not necessarily have general applicability. Conservation physiology, on the other hand, is more similar to medical treatments: after diagnosing the problem (e.g. fever), a treatment is applied (e.g. a non-steroidal medication), which is then followed by a search for the cause (e.g. a viral disease). Once the ultimate cause is known, it can be addressed directly (e.g. with an anti-viral and vaccination). The causes often have a relatively broad generality in their effect, so that implementing remedial actions focusing on causes is more effective than focusing on symptoms.

Many, if not most, conservation problems arise from environmental change brought about by human activity, such as climate change, deforestation, pollution, etc. These environmental signals interact with physiological receivers so that physiology is at the interface between the environment and individual organisms. Physiological responses to environmental change represent the mechanism, and therefore elucidate the biological cause, underlying the conservation problem. Ecological responses at the population, species, community and ecosystem levels are flow-on symptoms from the physiological causes (Coristine *et al.*, 2014). For example, in a basic population growth model, the rate of population increase depends on the thermal sensitivity of individual energy assimilation and growth (Savage *et al.*, 2004). Even in cases where population declines are caused by overharvesting such as for many fisheries (Gaines *et al.*, 2018), planning and implementing effective conservation action is contingent on an assessment of physiological responses—in this case, the thermal sensitivities of energetics and growth that determine population size (Savage *et al.*, 2004). Similar arguments can be made for habitat restoration, reintroductions and species distribution models, for example, all of which would need to understand physiological sensitivities to assess the suitability of habitats that are the focus of the conservation intervention or prediction. In these cases, physiological knowledge alone does not achieve conservation, but the success of conservation intervention without physiological knowledge can be unpredictable. To be clear, neither the resources nor time always exist to identify mechanisms, and in some cases, the threat is so evident (e.g. removal of habitat via land use change) that the conservation action is obvious. Yet, with a growing number of interacting stressors (e.g. with climate change as a threat multiplier), mechanisms will become more important to ensure evidence-informed decision-making (Cooke *et al.*, 2023). A synthesis where physiology, ecology, behaviour and other relevant fields (e.g. genetics) play a part can therefore help target conservation actions for maximum benefit.

There is also a growing evidence base that physiological data collected from individuals can indeed be scaled to population-level processes. When reviewed by Bergman *et al.* (2019), 21 studies were identified that simultaneously linked physiological information to environmental change and demographic responses. For example, telomere length could be used as a predictor of extinction risk under climate change for common lizards (*Zootoca vivipara*) (Dupoué *et al.*, 2017). Faecal androgen and glucocorticoid measurements have also been used to predict population decline under reduced food availability (grass abundance) in Cape Mountain zebra (*Equus zebra zebra*) (Lea *et al.*, 2017). The physiological metrics, taxonomic groups and environmental contexts varied across the studies identified by Bergman *et al.* (2019), with some bias towards vertebrates (particularly mammals) and physiological metrics that fall under the umbrella of ‘stress physiology’ (Bergman *et al.*, 2019). However, more investigations have been amassing that provide support for the predictive capacity of monitoring diverse types of physiology in other taxonomic groups (Ames *et al.*, 2020). Burford *et al.* (2022) showed that populations of California market squid (*Doryteuthis opalescens*) that form large populations due to competitive release undergo range expansions that are linked to an increase in temperature coupled with lower oxygen availability constraining aerobic activity. They further connected poleward range expansion to changes in body size that can have cascading trophic effects, with potential disruptions for resident species. This work highlighted the value of linking ecological processes with physiological information for predicting how marine ectotherms will respond to a changing climate and the associated ways in which communities will reorganize (Burford *et al.*, 2022).

While there are concrete examples that support the connection between individual physiological variation and demographic outcomes, this research avenue remains a pressing objective in conservation (specifically in the context of anthropogenic disturbance; Cooke *et al.*, 2021a). In the marine realm, a notable advance has occurred in the form of development of robust statistical approaches designed specifically to model how conservation-relevant changes in individual physiology scale to population-level effects (e.g. PCoD, Population Consequences of Disturbance; and PCoMS, Population Consequences of Multiple Stressors; Thomas *et al.*, 2025). Originally developed in response to National Research Council (2003) and National Academies of Sciences (2017) reports and workshops focused on effects of anthropogenic ocean noise, these PCoD and PCoMS models have since been broadened to incorporate multiple other environmental and anthropogenic impacts, and have been applied successfully to several long-lived marine taxa (e.g. gray whale, *Eschrichtius robustus*, Pirotta *et al.*, 2023; northern elephant seal, *Mirounga angustirostris*, North Atlantic right whale, *Eubalaena glacialis,* common bottlenose dolphin, *Tursiops truncatus*, Thomas *et al.*, 2025). Such modelling approaches are ripe for extension to terrestrial systems. With increasing attention to macrophysiology (e.g. trait-based approaches), landscape physiology, consideration of interspecific interactions, investigations across the lab-field continuum, modelling approaches and the increasing measurement of multiple physiological metrics simultaneously, the gap between individual and population levels can continue to close (Ames *et al.*, 2020; Binning *et al.*, 2025).

### Baseline (i.e. control) data are too sparse

Many studies are focused on assessing the change in a trait in relation to baseline data as an indicator of changes at the individual or population level. Having a reliable baseline is critical in experimental setups where a manipulation takes place and some set of responses is measured. But what are baseline data in the context of conservation science? This question is one that conservation scientists constantly struggle with (Monsarrat *et al.*, 2019; Rodrigues *et al.*, 2019), and not just in the physiological context. This challenge has been particularly true in the context of environmental pollution, and how physiological metrics (along with others) have been used to understand sublethal effects on biota . Specifically, where does one go to assess a pollution-free population? And how do we account for shifting baselines (i.e. the concept that the definition of what constitutes ‘natural’ conditions becomes degraded gradually over time due to lack of knowledge or experience; Pauly, 1995)? Many argue that for most contaminants, the baseline should be none, with the only exceptions being those compounds that are present in the environment at some concentration (e.g. mercury and cadmium, which are naturally occurring elements, some pyrogenic polycyclic aromatic hydrocarbons that are produced by forest fires). 

In the past few decades, much has been learned about the effects of oil on marine ecosystems through the use of physiological metrics, despite the absence of true ‘baseline’ data. Specifically, the Exxon-Valdez oil spill in Alaska in 1989, the Treasure oil spill in South Africa in 2000 and most recently the Deepwater Horizon spill in the Gulf of Mexico in 2010 have all led to an increased number of studies that have examined the effects of oil on marine ecosystems, from invertebrates to birds. Exposure to oil in the marine environment has demonstrably negative effects on marine species in a number of ways, including the ‘usual suspects’—direct fouling and mortality (Cadiou *et al.*, 2004; Alonso-Alvarez *et al.*, 2007), lower survival from reduced body mass (Paruk *et al.*, 2016) and reduced breeding success in the years following a spill (Barham *et al.*, 2007). But increasingly, physiological indicators are being used to assess the impacts of oil on marine organisms such as oxidative stress, haematocrit and blood chemistry, cardiac function, stress hormones and immune function (Takeshita *et al.*, 2021). Where oil spills have affected marine species, research assessing the impacts of oil on marine ecosystems would ideally rely on an oiled versus unoiled area research design. Because oil/fuel pollution at some level is present almost everywhere on the planet in the marine environment, experimental designs more often can only pair oil-affected areas with regions outside of the known spill zone (Iverson and Esler, 2010; Velando *et al.*, 2010). For example, Trust *et al.* (2000) found that the Cytochrome P450 1A induction was higher in two seabird species in an oiled area versus a reference area where no acute spill had occurred. More recently, some strategic environmental assessments of the potential impacts of oil pollution have carried out toxicogenomic and metabolomic studies in regions prior to acute spill or building events, but these studies demonstrate that even in remote regions where ‘baseline’ levels should be low, the best we can achieve is understanding the current benchmark levels (Provencher *et al.*, 2021; Zahaby *et al.*, 2021; Sarma *et al.*, 2022).

As a way to recognize that in many cases, the baseline state, or baseline data on a physiological trait, is beyond our reach, more and more researchers are comparing physiological readings when environmental conditions change to a reference state or benchmark level. Within this framing, the idea that altered states can only be compared to some historic baseline data can be put aside, with discussions focused on how appropriate benchmark sites and data should be considered. In conservation physiology, we are also observing the generation of reference ranges (or reference intervals), which are common in veterinary medicine. For example, Nieto-Claudín *et al.* (2021) determined haematological and biochemical reference ranges for critically endangered western Santa Cruz Galapágos tortoise (*Chelonoidis porteri*), and Valle *et al.* (2020) did the same for Galápagos shearwaters (*Puffinus subalaris*). Those reference ranges can be used to interpret physiological samples collected from those types of animals in the future to assess health status. In some cases, reference ranges can also be developed for past populations retrospectively via analyses of long-lived biomarkers in certain types of archival or museum specimens (e.g. keratin tissues such as fur or baleen; Bechshøft *et al.*, 2013; Shuttleworth *et al.*, 2025). With greater application of this pragmatic approach, there lies great opportunity to interpret physiological changes across spatial and temporal scales.

### It is difficult to develop general understanding in conservation physiology because of system complexity and diversity

One key challenge in conservation physiology research is the complexity of ecological systems and the challenges of modelling such intricate systems, which could hamper the ability of the field to yield new, generalizable patterns. For example, marine environments are intricate and influenced by numerous interacting factors, such as temperature and oxygen fluctuations, ocean acidification, turbidity and other anthropogenic disturbances. While researchers aim to uncover cause-and-effect relationships between physiological responses and environmental stressors, the multitude of variables often makes it challenging to establish definitive conclusions. For example, studies on coral reef fishes have demonstrated that physiological responses to stressors can vary depending on life stage, habitat and the specific stressor being examined (Rummer *et al.*, 2016; Johansen *et al.*, 2021; Rummer and Illing, 2022). However, there are also key approaches in conservation physiology that are founded on similarities that exist across organisms. Dynamic energy budget (DEB) theory relies on the underlying metabolic organization of cells that applies universally across species, allowing for the creation of DEB models for thousands of species and greater potential for extrapolating knowledge from one species to another (Lavaud *et al.*, 2021). Such approaches facilitate greater understanding of distribution shifts, demographic changes and other responses to anthropogenic change over wider spatial and temporal scales that can then be used to design networks of protected areas, identify candidate areas for restoration, plan reintroduction or assisted colonization programmes or limit invasive pest damage, among other applications (Evans *et al.*, 2015).

Conservation physiology can already offer more than just confirmatory insights. There is mounting evidence that a consideration of physiology can provide insight into broad-scale patterns that are relevant to conservation science. For example, a review by Funk (2013) illustrated that invasive plant species generally have higher root to shoot biomass ratio in arid environments, lower root to shoot biomass ratio in systems limited by light and lower leaf construction costs and higher photosynthetic energy use efficiency in light-limited systems. These physiological patterns indicate that plants invading low-resource environments possess traits related to resource use efficiency and/or conservation, thereby pointing to specific control methods (e.g. manual weeding, planting functionally similar native species, herbicide treatment or controlled burns rather than mowing or reducing resource availability) (Funk, 2013). Macrophysiological patterns have also been used to call for improved biosecurity protocols. The Intergovernmental Science-Policy Platform on Biodiversity and Ecosystem Services Invasive Alien Species Assessment incorporated information from multiple research outputs showing that terrestrial invasive invertebrate species have greater thermal tolerances, higher desiccation resistance and faster growth rates in comparison to native species (IPBES, 2021).

To enhance the reliability of conservation physiology research, a multi-faceted approach that works to bridge the lab/field divide and considers the complexities of ecological systems should be embraced (Binning *et al.*, 2025). More specifically, environmental complexity could be incrementally increased in laboratory systems or fluctuating exposures to environmental conditions could be incorporated, as informed by field data or observatories (Binning *et al.*, 2025). Mesocosms, longitudinal field studies and well-informed mathematical modelling approaches can all also contribute to bridging the lab/field divide to find a balance between experimental control and environmental complexity (Binning *et al.*, 2025). Factors such as acclimation, physiological and behavioural plasticity (over developmental and within as well as across generational timescales) and species interactions may significantly influence physiological outcomes in the wild, requiring their consideration in models aiming to apply findings more generally (Bhat *et al.*, 2015; Donelson *et al.*, 2018; Fan *et al.*, 2021). The application of DEB modelling to plants is another area with a great deal of unrealized potential that, with attention, could predict diversity shifts under climate change that are of great conservation concern (Russo *et al.*, 2022). It is further valuable to identify when modelling approaches that were robust in one species are more variable or less reliable in others, with particular emphasis on the mechanisms that lead to this variation. For example, Fogelman *et al.* (2025) examined the utility of a cellular energy allocation model for assessing how juvenile mussels respond to food limitation-induced stress. Although cellular energy allocation had been used as a holistic stress biomarker in other organisms, the authors did not find this to be the case in mussels and related this to the tendency of bivalves to reduce feeding and overall energy consumption under periods of stress (Fogelman *et al.*, 2025). Publishing these limitations encourages the application of tools in scenarios where they will be most useful, and helps to create a better picture of how generalizable methods can be. Sensitivity analyses will further help to determine which model parameters impart the greatest uncertainty in predicted outcomes and therefore identify the most pressing research requirements to maximize model performance (Pirotta, 2022). Finally, growing open-source resources and providing opportunities for training researchers and practitioners to learn how to use physiological data to inform models focused on conservation outcomes also has high potential to improve applicability (Binning *et al.*, 2025).

### Practitioners and decision-makers do not care about physiology

One of the challenges to better integrating physiology with practical conservation is the perception of some conservation practitioners that physiology is an abstract science. Nearly 30 years after a seminal agenda for conservation biology was laid out (Caughley, 1994), the discipline remains reactionary, heavily biassed towards the conservation of small populations (Caughley, 1994) and strapped for cash (Waldron *et al.*, 2013). Against this backdrop, it is hardly surprising that many people actively engaged with conservation see much greater value for money in ‘tangible’ conservation activity, such as reintroductions or translocations, or even just population monitoring, because this is money spent working directly with populations of threatened species and is inherently satisfying. In comparison with knowing how population sizes change over time, an understanding of physiology is often seen as abstract, and not informative of improved conservation outcomes (Cooke and O'Connor, 2010). Simply documenting a change in population abundance, size or sex structure, or the structure of communities, fails to identify the causal mechanism behind population declines or range collapse. That is where physiology truly delivers. Mechanisms matter in conservation if limited resources are to be directed towards the true cause/driver of a problem (Cooke *et al.*, 2023). Given that nature is complex, physiology can both reveal where efforts should likely be focused and then yield experimental studies to establish cause–effect relationships. Cause–effect relationships are ideal for providing regulatory guidance (e.g. related to types of land use change, pollution limits). Moreover, in the context of climate change (or other stressors), physiological knowledge can be used to develop predictive tools that managers can deploy in conservation planning, which may focus on strategic decision-making around protected areas (Seebacher and Franklin, 2012). Given the need for future-proof management actions, environmental managers are increasingly turning to models that are parameterized with physiological data (Zurell *et al.*, 2022; Tourinho and Vale, 2023). It is important to note that we do not attempt to argue that physiological information will be necessary or applicable to every conservation endeavour. We do, however, propose that it is useful to consider it regularly as an established tool in the conservation toolbox, and we provide a number of examples below that highlight how it has contributed to conservation action.

Physiology has directly informed captive breeding of herpetofauna. Captive breeding programmes lie at the heart of some of the most intensive management for conservation (Rahbek, 1993; Philippart, 1995). The understanding required to optimize a captive breeding programme, however, is essentially endocrinological and physiological. While there are many examples globally, recent advances in reptiles and amphibians demonstrate the fact well. The southern corroboree frog (*Pseudophryne corroboree*) is a critically endangered Australian frog, confined to only small populations in subalpine habitat of Kosciuszko National Park (Osborne, 1991; Hero *et al.*, 2006). Assisted reproductive technology can be a powerful component of a captive breeding programme, but in the case of anurans, it requires deep understanding of how to extract viable sperm and ova. Endocrinological research was fundamental in understanding how to best extract this material for assisted reproduction (Byrne and Silla, 2010), forming the basis of protocols in the National Recovery Plan for the species (Hunter, 2012). A similar study of reproductive cycling in Western Swamp Tortoises (*Pseudemydura umbrina*) was fundamental to developing a captive breeding programme (Kuchling and Bradshaw, 1993). The central importance of physiology in captive breeding and *ex situ* conservation is not confined to animals, either, with physiological responses recognized among the most critical ones to understand by seed biologists for application to ecological restoration (Dalziell *et al.*, 2022).

In most cases, physiological information will not be used in isolation; it will be combined with other forms of evidence (e.g. ecological data) to develop better-informed conservation approaches. Koalas provide a useful example where physiology has informed a number of ‘rescue’ interventions. Koalas are Australia’s national heritage wildlife species, and their wild numbers have significantly plummeted over the past few decades. Conservation physiology tools such as minimally invasive hormone monitoring methods have been used to demonstrate that koalas are stressed by exposure to human-induced disturbances, especially habitat loss, vehicle trauma and dog attacks (Narayan, 2019; Charalambous and Narayan, 2020). The International Fund for Animal Welfare (IFAW) used scientific evidence for Governmental deliberation on the change in conservation status of koalas. IFAW highlighted that stress is a major factor impacting koalas, and IFAW cited physiological research (Narayan, 2019; Narayan and Vanderneut, 2019) in the state government enquiry into koala populations and habitat in New South Wales, Australia (IFAW, 2019). IFAW underscored that evidence using conservation physiology methods should be applied in future policy approaches, ensuring that the long-term harm to individual animals and thus the wider population is considered in every planning project that involves removal of koala habitat and mitigation measures implemented that will truly protect koalas. In the key recommendations that can be guided by conservation physiology tools, IFAW requested a wildlife-friendly approach to urban planning adopted as a mandatory requirement to ensure that plans incorporate dedicated green spaces and wildlife corridors suitable for koalas and other wildlife, including designated wildlife crossings to ensure safe passage across roads. Koala rescuers are aware of the potential stress generated by koalas during wild rescues, and protocols are in place for best practice when attempting to capture and handle wild koalas (Flanagan, 2019).

Improving captive breeding protocols and monitoring hormonal data to delineate threats are some of the longest standing, single-species applications of conservation physiology (Bainbridge and Jabbour, 1998; Wikelski and Cooke, 2006; Narayan, 2013). However, there is also value in considering physiology in contexts where it has historically been underrepresented. For example, many conservation programmes aim to conserve communities, entire ecosystems and/or ecosystem services. While perhaps not as intuitive, physiological information has also been applicable to these scenarios. Many communities contain keystone species, including those that are necessary as ecological service providers whose presence and health are relevant to the overall conservation of an ecosystem. For example, in the context of the conservation of pollinator populations, combining landscape ecology with physiology can provide information on the broad-scale floral resource availability required to support colony growth and productivity (Alaux *et al.*, 2017). Through measurements of fat body mass and vitellogenin levels in honey bees (*Apis mellifera*), Alaux *et al.* (2017) determined that the presence of semi-natural habitats leads to an increase in pollen diet diversity and overall pollinator health, with downstream benefits for overwintering survival. These results inform conservation schemes aimed at supporting pollinators at the landscape level, with the authors suggesting that artificial bee pastures could be created as complementary management measures to semi-natural habitat protection (Alaux *et al.*, 2017).

Encouraging populations to reassemble following disturbance requires reestablishment of habitats that support their abiotic and biotic requirements, which can be ascertained through physiological studies. Pollinators again provide a meaningful example. Tomlinson *et al.* (2018) used a topoclimatic model to predict habitat suitability and energetic expenditure for four major hymenopteran pollinator species across a ~150 km^2^ restoration area in Western Australia. Their results indicate that restoration techniques will need to incorporate the planned provision of nutritional resources to ensure the return of insect pollination networks in these novel ecosystems (see further discussion below; Tomlinson *et al.*, 2018). Attention to physiological function has also proved helpful to planning when considering the restoration of native plant communities following the closure of mining operations. Within a biodiversity hotspot in southwest Australia, previous restoration monitoring had indicated seedling mortality rates as high as 90% during seasonal drought periods, with the underlying causes unknown (Burrows, 1986; Enright and Lamont, 1992; Rokich, 1999). Physiological performance (e.g. stomatal conductance, nutrient concentration) measures of multiple *Banksia* species seedlings indicated that soil amendment (native sourced-mulch) could improve capacity for tolerating severe drought stress (Benigno *et al.*, 2013). Together, morpho-physiological data indicated that seedling function can be better supported in these restoration landscapes to promote establishment of plant communities essential to overall restoration success (Benigno *et al.*, 2013). It is important to restate that we acknowledge that there will be scenarios where physiological information is not required to perform strong conservation work, but our goal here is to show that its applications are diverse and can indeed be relevant to practitioners and decision-makers.

### Conservation physiology is not for fundamental scientists

As we have stated above, conservation actions are often based on the observation of population-level changes and are made at the level of the species, community or ecosystem (Cooke and O'Connor, 2010). Therefore, scientists interested in a mechanistic understanding of a process with a focus at the cellular, molecular or organ level may feel farthest away from the conservation action and thus might be apprehensive to link their findings to population or ecosystem-level changes. However, fundamental science can provide physiological explanations that can be particularly important in identifying threats, predicting change and linking physiological processes to observed population declines based on the conservation physiology framework (Coristine *et al.*, 2014). In some cases, the physiological explanation has been used to effectively manage populations, while in other cases, this information can be incorporated into models or used by other scientists to provide the link to population-level outcomes. It is noteworthy that many researchers with expertise in conservation physiology were trained in fundamental comparative physiology; a core mechanistic physiology training has allowed many researchers and practitioners in the field to make contributions due to the fundamental understanding of physiology and a desire to do applied solution-based science.

Physiological mechanisms can provide explanations that can sometimes be generalized to many animals within an ecosystem and give insightful information on the effects of stressors. However, understanding the variation in the sensitivity of a species, individuals and even of different life stages to a particular stressor can inform which species, individual or life stage within a community or ecosystem is the most vulnerable and highlight those species that need immediate attention. For example, the mechanistic understanding of the cardiovascular physiology of migrating sockeye salmon (*Oncorhynchus nerka*) was used to explain why there was a high mortality of adult salmon when Fraser River temperatures are high during the summer (Eliason *et al.*, 2013). This result was achieved by measuring several steps in the oxygen transport cascade (Eliason *et al.*, 2013) to show that it was not the ability to extract oxygen at the gills as previously proposed (Brett, 1971) that causes this high mortality. Instead, the hearts of these fish did not function properly at temperatures above the optimal temperatures, leading to cardiac collapse and therefore death (Eliason *et al.*, 2013). Furthermore, measuring the cardiorespiratory function of several sockeye populations in the Fraser River revealed population level differences correlated with historical temperature profiles that different populations encountered during their migration (Eliason *et al.*, 2011). Finally, it became apparent that all the sockeye salmon populations in the Fraser River were experiencing higher than optimal temperatures and are threatened by rising temperatures and temperature extremes due to climate change (Cooke *et al.*, 2012). These findings are important to our fundamental understanding of cardiorespiratory function under changing temperature regimes. Simultaneously, these results have been used by the Pacific Salmon Commission to decide on catch allotment in order to balance conservation of salmon with harvest. As a result of this mechanistic approach to physiology, similar work is now underway in Oregon to determine the vulnerability of different populations of fish to summer maximum water temperatures and future predicted temperature increases (Anlauf-Dunn *et al.*, 2022). These results are being used by the Department of Fish and Wildlife to determine which species need to be prioritized for habitat rehabilitation and to determine fishing regulations in order to manage their populations effectively in the face of climate change.

Although not all contributions of fundamental science have been as successful as the examples above at being included into policy or management decisions, there are many more examples of how fundamental science can contribute to conservation efforts through a better understanding of the ultimate cause of a stressor and identifying threats. For example, a decrease in pH lowers the olfactory sensitivity of fish and invertebrates to various odorants, whether in marine (Porteus *et al.*, 2018; Velez *et al.*, 2019) or freshwater environments (Moore, 1994). This consequence is partially due to the effects of the pH on the odorant molecule itself (Roggatz *et al.*, 2016); the increase in proton concentration associated with a decrease in pH affects the protonation of odorant molecules (Roggatz *et al.*, 2016), which can decrease the probability these will bind to their receptors and has implications for key ecological functions of aquatic organisms. This mechanistic understanding of how biomolecules can be affected by a change in ocean pH has also led to the prediction that the toxicity of paralytic neurotoxins will increase in the future due to the intensification of ocean acidification and could result in an increased incidence of paralytic shellfish poisoning in the near future (Roggatz *et al.*, 2019). A detailed look at many conservation physiology endeavours reveals a foundation in the questions, concepts and theories of physiological ecology and evolution that, in turn, allow better interpretation and application of results in applied settings. Consequently, individuals working on primarily fundamental scientific questions have great potential to refine the details of the application of physiological tools to conservation challenges, and there are opportunities for connecting with applied scientists to do so (see the next section).

### Conservation physiology cannot contribute to solving major conservation challenges

For some, conservation physiology as a discipline is viewed purely as an area of fundamental scholarly inquiry rather than an applied field that makes meaningful contributions to solving conservation problems (Madliger *et al.*, 2021a). It is true that some conservation physiology research does not immediately or directly benefit conservation, which is expected. In many ways, conservation physiology is best viewed along the fundamental-applied continuum with valid and important work being done along the entirety of that continuum. Fundamental work is needed to establish relationships and understand how, for example, different stressors influence organismal function (Courchamp *et al.*, 2015). Such work may not be immediately operational, yet can be foundational for future research, and, as discussed in the previous section, can be an important avenue for interested scientists to begin connecting their fundamental research to applied scenarios. Further, follow-up studies that test different mitigation strategies may yield management options that can be implemented by practitioners. Over the last few decades, there have been increasing examples of how conservation physiology has contributed to conservation success (as summarized in Madliger *et al.*, 2016 and Cooke *et al.*, 2021a).

Even seemingly abstract aspects of physiology can be valuable in guiding conservation activity. Land use change and degradation often result in fundamental changes to the abiotic environment, either cooling the environment down (Garcia and Clusella-Trullas, 2019) or creating more open, exposed habitats. In the southwest of Western Australia, clearing of native *Banksia* woodland has resulted in heavily degraded peri-urban wastelands, dominated by invasive grasses that surround isolated remnant pockets of native vegetation (Ritchie *et al.*, 2021). These exposed environments represent barriers to dispersal by insect pollinators, but not because these landscapes are too hot. Indeed, the temperatures in these exposed landscapes are well within the tolerance limits of most key native hymenopteran pollinators in the region (Tomlinson *et al.*, 2015). However, the high temperatures impose energetic costs on any bees that might disperse between remnants, and modelling suggests that these costs underpin the inability of bees to disperse between pockets of native bushland (Tomlinson *et al.*, 2018). The practical guidance that resulted from this was the establishment of nectar-rich plantings to act as stepping stones between native remnant patches to facilitate restoration programmes in the region. The practical value of such insights to ecological restoration, garnered from physiological studies, has recently been the focus of two reviews (Tomlinson *et al.*, 2021; Valliere *et al.*, 2022) and provides strong refutation that physiological studies cannot guide concrete conservation practices.

Even when knowledge exists that could be embraced and operationalized in conservation decision-making, there are a number of reasons why that might not occur. There is a well-known gap between science and action (variously termed the knowledge–action gap, the theory–practice gap, the research–application gap, etc.; Cooke *et al.*, 2021b). The knowledge–action gap recognizes the reality that even when there is evidence that could potentially transform the decisions being made by practitioners, there are a variety of barriers to its use. The barriers are wide ranging and have been well studied in the conservation and environment space (reviewed in Cook *et al.*, 2013) and presumably are pervasive in conservation physiology. One potential barrier is accessibility. Much of the available science is behind paywalls, making it difficult for practitioners to access material (Roche *et al.*, 2022). Fortunately, that is not the case for the journal *Conservation Physiology*, which is open access.

Sometimes the characteristics of the evidence base may themselves also serve as a barrier. For example, a single, one-off empirical study would not be considered as reliable or robust as a synthesis of relevant evidence (e.g. a systematic review, meta-analysis), which assembles relevant literature and provides signals about the overall pattern emerging from a body of research (Pullin *et al.*, 2020). Fortunately, we are seeing more of these syntheses in the conservation physiology space that provide practitioners with actionable knowledge. For example, a review on the physiology of elasmobranch bycatch provides information on mitigation strategies (Jordan *et al.*, 2013), while a review on the physiology of conservation translocations identifies methods that are most likely to yield success (Tarszisz *et al.*, 2014). Providing practitioners with tools they can apply to their decision-making processes (e.g. bioenergetics models; Pirotta, 2022) is an example of another way to be relevant. Being relevant also means answering questions that are of interest to practitioners (Cook *et al.*, 2013; Kadykalo *et al.*, 2021). The best way to address that issue is through co-production where researchers and knowledge users work hand in hand to co-develop research questions, to secure funding and to conduct and interpret the research (Caro *et al.*, 2023). This approach ensures opportunities for knowledge exchange and is one of the most effective ways to do science in a way that will have impact on conservation solutions (Beier *et al.*, 2017). We are beginning to see examples of papers in *Conservation Physiology* co-authored by practitioners (Patterson *et al.*, 2016) as well as stakeholders (Lewandrowski *et al.*, 2024) and rightsholders. Moreover, a recent synthesis presented a series of case studies in conservation physiology from around the globe where co-production was embraced (Cooke *et al.*, 2025). A key message arising from Cooke *et al.* (2025) was that relationships and trust that are essential and inherent in co-production (done well!) helped to establish mutual understanding and overcome challenges such as the misconceptions identified in the current paper. In fact, one of the explicit benefits noted in that paper was the ability to learn and share experiences from different knowledge systems, lived experiences and disciplines to work collaboratively and efficiently towards solutions.

Lastly, we recognize that conservation challenges are complex—those with simple solutions are often not the most pressing challenges. In a landscape where conservation challenges are pressing and immediate, and there is not enough time or money to do all the work that is needed, there is a move towards expert knowledge synthesis to prioritize and leverage conservation actions of the most immediate benefit (Provencher *et al.*, 2023; Rooney *et al.*, 2023). These processes can use expert opinion across disciplines to fill knowledge to action needs in data-poor environments, including principles from physiological conservation studies within the larger conservation context. For example, expert opinion methods were used to understand how a novel strain of avian influenza may influence ducks in North America under different exposure levels, both at the individual and population level (Provencher *et al.*, 2023). Critical to these discussions was understanding the mechanisms of how disease can affect individuals and populations mechanistically in order to extrapolate to unknown scenarios, demonstrating further that conservation physiology can contribute to conservation implementation. Just like any conservation discipline, some investigations will have more immediate application value than others, but conservation physiology has a growing list of examples that illustrate it can indeed contribute to solving conservation challenges. In the next decade, we anticipate even more of this as the research community learns more about how to be relevant to practitioners and to narrow the knowledge-action gap.

## A starting point for more readily linking physiological data with conservation action

Conservation physiology is inherently a discipline-spanning area of research and practice (Cooke *et al.*, 2013). With that come challenges. Because conservation physiology is mission oriented, it differs from allied disciplines such as environmental and ecological physiology. Conservation physiology certainly leans on those (and other) disciplines for its foundations, but it is about understanding and solving problems related to conservation and sustainable resource management. Ensuring that science is relevant to end-users and is actionable remains a challenge (Cooke *et al.*, 2021b). Indeed, several of the misconceptions identified above are related to this topic. However, we are making inroads and equipping our community with the skills to be able to generate knowledge in manners that have the greatest potential to affect change and influence policy and practice. At the core is the need for more co-production models where researchers work hand in hand with decision-makers, stakeholders and rights holders to develop, conduct, interpret and apply evidence (Beier *et al.*, 2017; as has recently been discussed in the context of conservation physiology—see Cooke *et al.*, 2025). Another key is the need to engage in effective knowledge exchange with diverse and relevant partners. In many ways, this is adjunct to the concept of knowledge mobilization and recognizes that communication is not unidirectional but rather an exchange of ideas (Fazey *et al.*, 2013; Cooke *et al.*, 2021b). Part of this process is explicitly demonstrating the manner in which the conservation physiology toolbox can contribute to establish environmental and conservation management structures, such as recent efforts to integrate physiological approaches into each stage of the adaptive management loop (Tudor *et al.*, 2023). Effective knowledge exchange represents an obvious opportunity to address some of the misconceptions raised above. Specific to conservation physiology, early collaboration, identifying the most appropriate tools within (or outside of) the conservation physiology toolbox, translating physiological data into usable formats and linking it to management interventions and decisions show particular promise for making conservation physiology even more relevant. As a starting point for those considering the application of physiology to specific challenges, systems or contexts, we have provided a list of resources (Table 1). The listed reviews, meta-analyses and perspectives are focused on specific taxa or conservation challenges, rather than providing detailed overviews of individual physiological tools. In this way, readers can explore the full array of physiological information that is applicable to their topics of interest, and use these as a jumping-off point for diving deeper into the specifics of the tools that align with their goals.

**Figure 1:**
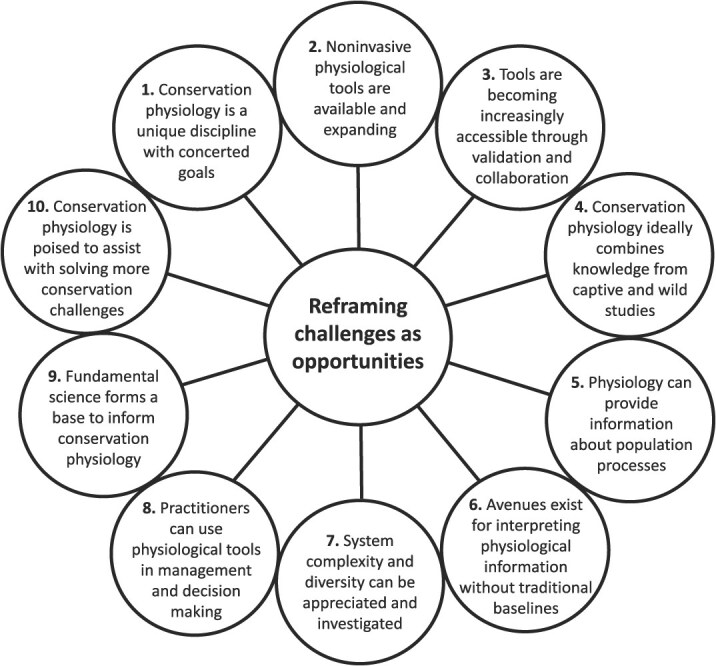
A restatement of 10 misconceptions in conservation physiology as opportunities to appreciate the growth and establishment of the field

**Table 1:** Selection of review, perspective and meta-analysis articles that deal with the application of physiological knowledge and tools within specific taxa or contexts of conservation

**Category**	**Subtopic**	**Selected publications**	**References**
Taxonomic groups	Amphibians	Physiology, environmental change and anuran conservation	Navas and Otani, 2007
		Physiological ecology and conservation of anuran amphibians	Navas *et al.*, 2016
		Integrating behaviour and physiology into strategies for amphibian conservation	Walls and Gabor, 2019
		Amphibian reproductive technologies: approaches and welfare considerations	Silla *et al.*, 2021
	Bees	Using physiology to better support wild bee conservation	Leroy *et al.*, 2023
	Fishes	Physiology can contribute to better understanding, management and conservation of coral reef fishes	Illing and Rummer, 2017
		Bridging disciplines to advance elasmobranch conservation: applications of physiological ecology	Lyons *et al.*, 2019
		Conservation physiology of freshwater fishes: an illustration of pressing questions and implications for management	Pleizier *et al.*, 2025
	Mammals	Overcoming the challenges of studying conservation physiology in large whales: a review of available methods	Hunt *et al.*, 2013
		Physiological thresholds in the context of marine mammal conservation	Acevedo-Whitehouse, 2019
		The use of non-invasive and minimally invasive methods in endocrinology for threatened mammalian species conservation	Kersey and Dehnhard, 2014
	Plants	Conservation physiology of plants	Van Kleunen, 2014
		Plant physiological indicators for optimizing conservation outcomes	Schönbeck *et al.*, 2023;
Threats	Climate change	From cells to coastlines: how can we use physiology to forecast the impacts of climate change?	Helmuth, 2009
		How can physiology best contribute to wildlife conservation in a warming world?	Seebacher *et al.*, 2023
	Disease and pathogens	Using physiology to understand climate-driven changes in disease and their implications for conservation	Rohr *et al.*, 2013
		Applied ecoimmunology: using immunological tools to improve conservation efforts in a changing world	Ohmer *et al.*, 2021
	Invasive species	The physiology of invasive plants in low-resource environments	Funk, 2013
		The role thermal physiology plays in species invasion	Kelley, 2014
		Improving science-based invasive species management with physiological knowledge, concepts and tools	Lennox *et al.*, 2015
Management applications	At-risk animal recovery planning	Conservation physiology can inform threat assessment and recovery planning processes for threatened species	Birnie-Gauvin *et al.*, 2017
		An assessment of the US endangered species act recovery plans: using physiology to support conservation	Mahoney *et al.*, 2018
		Physiology as a tool for at-risk animal recovery planning: an analysis of Canadian recovery strategies with global recommendations	Madliger *et al.*, 2022
	Ecological restoration	Ecological restoration and physiology: an overdue integration	Cooke and Suski, 2008
		Leveraging the value of conservation physiology for ecological restoration	Tomlinson *et al.*, 2021
		Restoration ecophysiology: an ecophysiological approach to improve restoration strategies and outcomes in severely disturbed landscapes	Valliere *et al.*, 2022
		Integrating animal physiology into the adaptive management of restored landscapes	Tudor *et al.*, 2023
	Fisheries management	Integrating physiology and life history to improve fisheries management and conservation	Young *et al.*, 2006
		Physiological biomarkers and fisheries management	Brosset *et al.*, 2021
	Forecasting risk	Trait-based approaches to conservation physiology: forecasting environmental change risks from the bottom up	Chown, 2012
		Defence mechanisms: the role of physiology in current and future environmental protection paradigms	Glover, 2018
	Policy	Making conservation physiology relevant to policy makers and conservation practitioners	Cooke and O'Connor, 2010
		Harnessing physiological research for smarter environmental policy	Dubuc *et al.*, 2025
	Translocations	Physiology in conservation translocations	Tarszisz *et al.*, 2014
Refinement and reflection	Scaling physiology to the population level	Scaling from individual physiological measures to population-level demographic change: case studies and future directions for conservation management	Bergman *et al.*, 2019
		Striving for population-level conservation: integrating physiology across the biological hierarchy	Ames *et al.*, 2020
	Success stories in conservation physiology	Success stories and emerging themes in conservation physiology	Madliger *et al.*, 2016
		The second warning to humanity: contributions and solutions from conservation physiology	Madliger *et al.*, 2021c
		Co-production and conservation physiology: outcomes, challenges and opportunities arising from reflections on diverse co-produced projects	Cooke *et al.*, 2025

This list offers a subset of the total literature and aims to provide readers with a starting point to examine the application of physiology within subfields that are pertinent to their work.

A mechanistic understanding can be important in providing the specific mode of action of a stressor at the organism level or below, and it has the potential to be used to identify the best solution for mitigation or to improve management practices. Sometimes the findings of fundamental scientists are not immediately useful for management applications, but could become so later. That observation featured prominently in some of the misconceptions. Fundamental science is more likely to be used in conservation or management decisions when these approaches are combined with historical or current environmental data and/or modelling to make predictions about future changes in an era of anthropogenic change. Therefore, fundamental science and scientists have a central contribution in the field of conservation physiology.

When reading this paper, it is important to recognize that the above misconceptions may be responsible for why practitioners (policy makers and managers) are hesitant to incorporate conservation physiology into management actions. In that sense, every misconception represents an explicit or implicit barrier. The upshot is that we were able to dispel those misconceptions using evidence and examples. In other words, what might appear as a barrier is in fact an opportunity that needs to be embraced (Figure 1). This paper is intended to open the eyes and minds of both conservation physiologists and end-users about what conservation physiology has to offer and how to realize those opportunities. Importantly, no one has to do it alone. Conservation physiology as a discipline is a cohesive group that behaves very much like a community of practice. Working collectively and collaboratively to achieve the common mission of conservation physiology will not only benefit the conservation physiology community but also benefit biodiversity and civil society.

Box 1: A subset of criticisms received as open-ended comments through an anonymous online survey of 468 scientists that have published research combining physiological approaches and conservation implications (data collected in 2016). Full methodology and results of the survey can be viewed in Madliger *et al.* (2021a).

- Physiology has not yet gained any status in conservation biology.
- I’m really not convinced that ‘Conservation Physiology’ should be considered a field. It seems like an unnecessary attempt to carve out a field that encompasses conservation, ecology and physiology.
- Conservation physiology is unknown to most conservation biologists and not considered important.
- [Conservation Physiology is] still too young of a field, and the techniques have not yet been proven to be particularly useful to conservation
- It seems to be a discipline that tries to subsume a number of pre-existing disciplines whereby there have been some ecosystem successes (i.e. restoration ecology).
- In my personal experience—the work I’ve done and that I’m familiar with through my colleagues, I’m not aware of any instance where physiological measurements have been the primary evidence used for a management/policy change.
- Conservation Physiology is viewed as pseudo-science without rigour and based only on conceptual thinking but little real application.
- The physiologists I work with rarely if ever consider population-level effects.
- As far as I can tell, this burden of proof (that the [physiological] variable is tied to population productivity) has rarely been achieved. In instances where there is a strong case that a physiological measurement is tied to fitness, its measurement may be too labour intensive, time-consuming, costly or technical for a conservation or resource management agency to integrate it into routine monitoring and assessment.
- Linking even simple physiological measurements to impacts on real populations is very difficult, and there are few precedents.
- I have found it hard to convince conservationists to let me do invasive procedures (implanting body temperature data loggers); I believe that this is because conservationists favour hands-off approaches and often think that my procedures are more damaging to the animal than they are in reality.
- There is a frequent disconnect with some ecologists/conservationists who think that physiologists are going to harm the animals.
- Short-sighted opinions that physiology is too invasive to be risked on endangered species means that funding is rare and restricted.
- Body tissue or fluid samples or physiological metrics taken from an animal that has been chased/caught and/or chemically immobilized are highly unlikely to play an important informative role in conservation biology.
- Lack of baseline physiological data is always a problem, and it often results in big time lags between getting data and applying it to a conservation plan/goal.
- Researchers often only start working with a group of organisms when those become vulnerable or start to decline in response to environmental change. In these cases, baseline data are almost always impossible to get.
- Physiological tools are not validated for use in conservation settings.
- The greatest challenge is demonstrating the benefit that physiological research can have for practical conservation problems.

## Data Availability

No data were generated or analysed during this work.
